# Hepatoprotective Polysaccharides from *Geranium wilfordii*: Purification, Structural Characterization, and Their Mechanism

**DOI:** 10.3390/molecules27113602

**Published:** 2022-06-03

**Authors:** Jia-Yi Feng, Yan-Qing Xie, Peng Zhang, Qian Zhou, Afsar Khan, Zhi-Hong Zhou, Xian-Song Xia, Lu Liu

**Affiliations:** 1 Yunnan Yunzhong Research Institute of Nutrition and Health, Yunnan University of Chinese Medicine, Kunming 650500, China; fjy15044976786@163.com (J.-Y.F.); 15096799906@163.com (Y.-Q.X.); zhp15887151484@163.com (P.Z.); 15911614191@163.com (Q.Z.); kmzhouzh@163.com (Z.-H.Z.); 2 Department of Chemistry, Abbottabad Campus, COMSATS University Islamabad, Abbottabad 22060, Pakistan; afsarhej@gmail.com

**Keywords:** *Geranium wilfordii*, polysaccharide, acute liver injury, hepatoprotective effect, anti-inflammatory activities

## Abstract

Traditional Chinese Medicine is generally used as a decoction to guard health. Many active ingredients in the decoction are chemical ingredients that are not usually paid attention to in phytochemical research, such as polysaccharides, etc. Based on research interest in Chinese herbal decoction, crude polysaccharides from *G. wilfordii* (GCP) were purified to obtain two relatively homogeneous polysaccharides, a neutral polysaccharide (GNP), and an acid polysaccharide (GAP) by various chromatographic separation methods, which were initially characterized by GC-MS, NMR, IR, and methylation analysis. Studies on the hepatoprotective activity of GCP in vivo showed that GCP might be a potential agent for the prevention and treatment of acute liver injury by inhibiting the secretion levels of ALT, AST, IL-6, IL-1*β*, TNF-*α*, and MDA expression levels, increasing SOD, and the GSH-Px activity value. Further, in vitro assays, GNP and GAP, decrease the inflammatory response by inhibiting the secretion of IL-6 and TNF-*α*, involved in the STAT1/T-bet signaling pathway.

## 1. Introduction

The liver is an extremely important solid organ, which is responsible for important physiological functions such as metabolism, excretion, and detoxification [1]. Acute liver injury (ALI), the main type of liver disease, has a mortality rate as high as about 20–40% [2]. ALI can initiate cascades of pathological processes, and further results in the development of liver fibrosis and even liver cancer [3]. At present, the incidence of ALI in China is increasing year by year, accounting for 15–30% of patients with fulminant liver failure [4], which is usually caused by toxic chemicals, drugs, or pathogen infections [5]. Oxidative stress and inflammation are the two major mechanisms underlying the pathogenesis of ALI [6,7]. Research is increasing to treat ALI by screening for ingredients with potent antioxidant and anti-inflammatory properties [8,9].

Animals with liver injury induced by CCl_4_ are typical experimental models that have been comprehensively applied in efficacy evaluation and pharmacological investigation of liver-protective drugs [10,11,12]. Additionally, the pathological mechanism of inflammation and apoptosis induced by CCl_4_ in the liver can better simulate acute chemical liver injury in humans [13]. As well as for screening of active compounds. CCl_4_ is metabolized by cytochrome P450 into the trichloromethyl free radical (CCl_3_) and trichloromethyl peroxyl free radical (CCl_3_OO), causing membrane lipid peroxidation [14]. Subsequently, the liver is induced to release the pro-inflammatory cytokines tumor necrosis factor-*α* (TNF-*α*), interleukin 6 (IL-6), and interleukin 1*β* (IL-1*β*) [15], which directly cause acute liver injury. Thus, CCl_4_-induced liver injury can be treated with anti-inflammatory and antioxidant supplementation [16].

Polysaccharides are high molecular carbohydrates, consisting of at least 10 monosaccharides linked by glycosidic bonds [17], and they are generally more likely to be the polar components in herbs and are mostly extracted with water, which is similar to the traditional Chinese medicine decoction used as medicine. Modern medicine, pharmacology, and food nutrition studies have shown that polysaccharides have many biological activities such as immunoregulatory [18,19] and antioxidative [20,21]. This also shows that polysaccharides are one of the most potentially bioactive ingredients, particularly due to their antioxidant, anti-inflammatory, and immunomodulatory activities [22]. In addition, the biological activity of polysaccharides can be affected by their structural properties [23]. However, modern phytochemical research mostly focuses on small or moderately polar molecules, and less attention is paid to polysaccharides in water extracts.

*Geranium wilfordii* is mostly used to treat tuberculosis fever, bruises, and rheumatism in folk medicine, distributed in Southwest China, in the Yunnan, Sichuan, and Guizhou Provinces [24]. *G. wilfordii* oral liquid is clinically used for the treatment of chronic hepatitis B, based on its good antioxidant and anti-inflammatory effects [25]. At present, we have not found a complete and fully explained research content on the structure and hepatoprotective activity of GCP. Thus, a preliminary study of GCP was carried out on the structural characterization of polysaccharides, their hepatoprotective activity, and their mechanism.

## 2. Results

### 2.1. Preparation and Characterization of GCP

The extraction yield of GCP was 12% w/w of the *G. wilfordii*. The total carbohydrate content of GCP was 46.05%, which was measured by phenol-H_2_SO_4_ assay [26]. In addition, the Coomassie brilliant blue method showed that the protein content in GCP was 3.8% [27].

### 2.2. Structural Characterization of GCP, GNP, and GAP

#### 2.2.1. Molecular Weights of GCP, GNP, and GAP

GCP showed two peaks from which the molecular weights were estimated to be approximately 1591 and 750.9 Da, respectively; GNP and GAP had the molecular weights of 3314 and 2651 Da, respectively, and the peak shapes of GNP and GAP were relatively symmetrical and uniform (Figure S1A–C).

#### 2.2.2. FT-IR Spectral Analysis

The FT-IR spectra of GCP, GNP, and GAP are shown in Figure S1D–F. GCP, GNP, and GAP all have strong -OH stretching vibrations absorption peak near 3400 cm^−1^; and weak C-H stretching vibration near 2930 cm^−1^ [28]_._ GCP and GAP have strong -OH bending vibrations near 1600 cm^−1^, and GNP has slightly weaker -OH bending vibration; GCP, GNP, and GAP are all characterized by C-O stretching vibration at 1215–1220 cm^−1^; and it is the absorption band of -OH variable angle vibration near 1032–1034 cm^−1^; GCP and GNP are presumed to show the characteristic band of *β*-glycosidic bond at 937 cm^−1^; GCP, GNP, and GAP show the characteristic absorption band of *α*-pyranose at 832 cm^−1^ [29].

#### 2.2.3. Monosaccharide Composition Analysis

As shown in Figure S1G,H, the GNP is a heteropolysaccharide mainly consisting of galactose (Gal), glucose (Glc), and arabinose (Ara) with molar percentages of 32.3, 25.01, and 16.83%, respectively. GAP was a heteropolysaccharide mainly consisting of Ara, Gal, and Glc with molar percentages of 35.24, 26.33, and 23.48%, respectively.

#### 2.2.4. Methylation Analysis

As shown in Tables S1 and S2, various types of bonds were identified in the GC-MS spectra of GNP, as well as GAP methylation. The main bond types in GNP are 1,4-Gal*p*, 1,2-Glc*p*, 1,5-Ara*f*, and 1-Glc*p*, accounting for 57.7% of the total methylated residues. The main bond types in GAP were 1,4-Gal*p*, 1-Ara*f*, 1-Gal*p*, and 1-Glc*p*, accounting for 47.1% of the total methylated residues, which were basically consistent with the monosaccharide composition results.

#### 2.2.5. NMR Analysis

The ^1^H- and ^13^C-NMR spectra of GNP and GAP are shown in Figure S2. In the ^1^H-NMR spectrum of GNP, six anomeric proton signals were observed at *δ*_H_: 5.19, 5.10, 4.76, 4.68, 4.48, and 4.44 (named A–F), while five anomeric carbon signals appeared at *δ*_C_ 104.0, 103.0, 101.0, 97.4, and 92.1 in the ^13^C-NMR spectrum. In the ^1^H-NMR spectrum of GAP, three anomeric proton signals were found at *δ*_H_: 5.00, 4.93, and 4.33 (named A′–C′), while three anomeric carbon signals appeared at *δ*_C_ 107.4, 107.0, and 103.6 in the ^13^C-NMR spectrum. The ^1^H- and ^13^C-NMR chemical shifts of GNP and GAP are summarized in Tables S3 and S4.

##### 2D-NMR Analysis of GNP

In the HSQC spectrum of GNP (Figure S2C), the cross peaks were found at *δ* 4.44/103.0 (A), 5.19/92.1 (B), and 4.68/98.9 (D), while the residues C, E, and F had incomplete chemical shifts, but could also be identified according to other signals.

In the ^1^H-NMR, residue A had an anomeric proton signal at *δ*_H_ 4.44 ppm (Figure S2A). Five cross-peaks at *δ*_H_ 4.44/3.78, 3.78/3.50, 3.50/4.10, 4.10/3.61, and 3.61/3.32 ppm were detected easily in the ^1^H-^1^H COSY spectrum (Figure S2D); hence, the H1–H6 of B were indicated. Based on the ^1^H-NMR assignment from the ^1^H-^1^H COSY spectrum for residue A, the carbon signals in the HSQC spectrum were easily assigned. The cross-peaks *δ* 4.44/103.0, 3.78/69.4, 3.50/77.2, 4.10/76.5, 3.61/73.0, and 3.32/61.0 ppm corresponded to H1/C1-H6/C6 of residue A. According to the methylation analysis results and compared with previous data, residue A was assigned as →1)-*α*-D-Gal*p*-(4→ [30].

For residue B, there was an anomeric proton signal at *δ*_H_ 5.19 ppm and a cross-peak at *δ* 5.19/92.1 ppm in the HSQC spectrum showed that the residue B was T-linked-Glc*p*. The ^1^H-NMR assignments were carried out through the ^1^H-^1^H COSY spectrum (Figure S2D), which were *δ*_H_ 3.80, 3.51, 3.24, and 3.55 ppm for H-2, H-3, H-4, H-5, while the correlation of H-6 was not found because its signal was near that of DMSO. The cross-peaks at *δ* 5.19/92.1, 3.80/74.2, 3.51/74.3, 3.24/71.1, and 3.66/73.0 ppm in the HSQC spectrum indicated the H1/C1-H5/C5 signals of residue B, while the signal of C-6 appeared at *δ*_C_ 61.2. The residue B was assigned as *α*-D-Glc*p* by the methylation analysis results and compared with previous data [31].

The cross-peaks *δ* 4.68/98.9 in the HSQC spectrum could be attributed to H1/C1 of the residue D, which might be a *β*-D-Glc*p*. The ^1^H-^1^H COSY cross-peaks were observed at *δ*_H_ 4.68/4.04, 4.04/4.30, 4.30/3.79, 3.79/3.52, and 3.52/3.43 ppm which suggested H1-H6 of residue D. The C2-C6 signals of residue D were accurately assigned from the HSQC spectrum. The chemical shifts of residue D were matched with previously published data [32], which suggested it as →1)-*β*-D-Glc*p*.

Although the C1 of residues C, E, and F were hard to be found in the HSQC spectrum, the existence of →1)-*α*-D-Ara*f*, →1)-*β*-D-Glc*p*-(4→ and →1)-*α*-D-Ara*f*-(5→ were proved according to other signals. Hence, the residues C, E, and F were matched by previously published data [33,34] and the methylation analysis results, which identified these three residues as →1)-*α*-D-Ara*f*, →1)-*β*-D-Glc*p*-(4→ and →1)-*α*-D-Ara*f*-(5→.

##### NMR Analysis of GAP

From the HSQC spectrum (Figure S2C′), the anomeric carbon signals at chemical shifts *δ*_C_ 107.4, 107.0, and 103.6 ppm cross linked to the anomeric proton signals at *δ*_H_ 4.94 (A′), 5.00 (B′), and 4.45 (C′), respectively.

In the case of residue A′, the H-2 was determined from the cross-peaks *δ*_H_ 4.33/3.32 ppm in the ^1^H-^1^H COSY spectrum (Figure S2D′). The other cross peaks were found in the ^1^H-^1^H COSY spectrum at *δ*_H_ 3.32/3.53, 3.53/3.72, 3.72/3.57, and 3.57/3.70 ppm, suggesting the H1-H6 of residue A′. The cross-peaks at *δ* 4.45/103.6, 3.32/74.3, 3.53/76.7, 3.72/72.5, 3.57/74.3, and 3.70/61.0 ppm in the HSQC spectrum indicated the H1/C1-H6/C6 signals of residue A′. According to the methylation analysis results and previously published data [35], the residue A′ was identified as →1)-*β*-D-Glc*p*.

The residues B′ and C′ were analyzed in the same way. The cross-peaks at *δ* 5.00/107.0 and 4.94/107.4 ppm in the HSQC spectrum were attributed to C-1 of *α*-D-Ara*f* and *β*-D-Gal*p*. According to ^1^H-^1^H COSY and HSQC spectra, the H1/C1-H6/C6 of residues B′ and C′ were fully inferred. The results were compared with the previous date and from methylation analysis, the residue B′ was assigned as →1)-*α*-D-Ara*f*, the residue C′ was suggested to be →1)-*β*-D-Gal*p*-(4→ [33,36].

### 2.3. GCP Ameliorates CCl_4_-Induced Hepatotoxicity

Table 1 shows the liver and spleen index of mice. The CCl_4_-treated mice significantly gained liver and spleen index in comparison with the normal group (*p* < 0.001, *p* < 0.0001). At the doses of 300 and 600 mg/kg body weight (bw) GCP administration significantly decreased the liver and spleen index compared with CCl_4_ group (*p* < 0.05, *p* < 0.01, *p* < 0.001, *p* < 0.0001). As shown in Table 1, there was also observed a similar effect with the positive drug, biphenyl diester (150 mg/kg bw) in mice.

**Figure 1:**
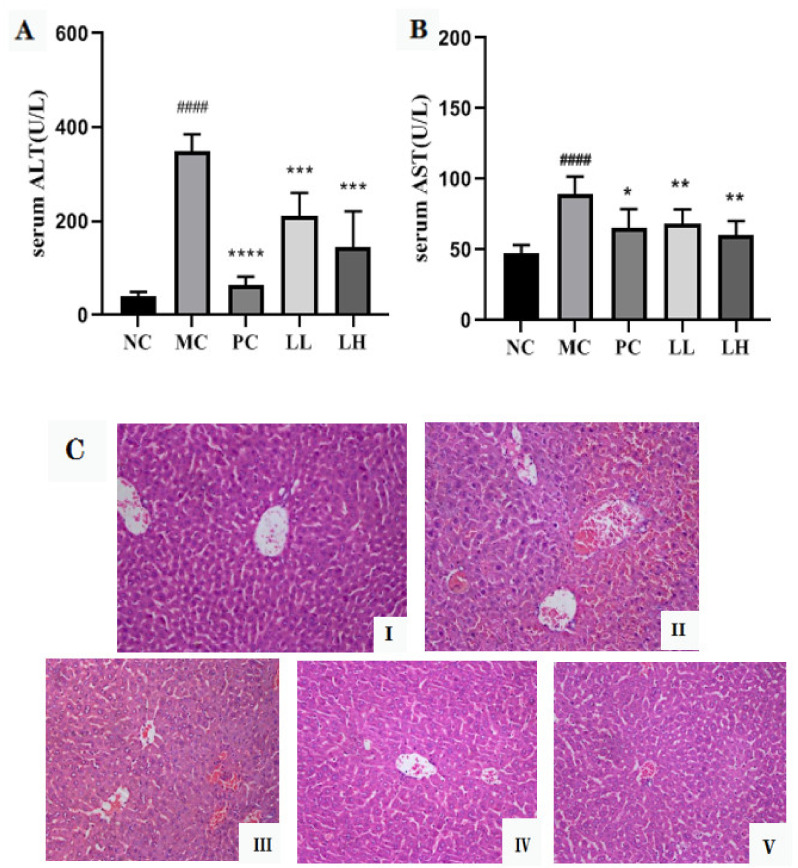
Effects of GCP on the levels of ALT (**A**), AST (**B**), and histopathological examination by H & E staining (20×) (**C**) against CCl_4_-induced liver injury in mice. Group I: normal control, Group II: CCl_4_ model control, Group III: positive drug group, Group IV: GCP low dose control (300 mg/kg), Group V: GCP high dose control (600 mg/kg); values are presented as mean ± SD for eight mice in each group. #### *p* < 0.0001, vs. normal control group. * *p* < 0.05, ** *p* < 0.01, *** *p* < 0.001, **** *p* < 0.0001, vs. model control group.

As shown in Figure 1A,B, the serum activities of ALT and AST in CCl_4_-treated mice were significantly increased (*p* < 0.0001), then compared to those of the normal control group. However, pretreatment with GCP significantly (*p* < 0.001, *p* < 0.0001) and dose-dependently lowered the ALT and AST activities as compared to the model control group. Moreover, the positive drug group also significantly reduced the activities of ALT and AST (*p* < 0.05).

H & E-stained sections are shown in Figure 1C. There was no abnormal appearance in the liver of mice in the normal control group (Figure 1C(I)). Intoxication of CCl_4_ caused severe hepatocyte necrosis, condensed nuclei, and massive inflammatory cell infiltration (Figure 1C(II)). However, the GCP and biphenyl diester group could ameliorate CCl_4_-induced liver damage, as demonstrated by necrotic areas with markedly decreased and slight inflammatory cell infiltration (Figure 1C(III–V)).

### 2.4. GCP Reduced the Expressions of Pro-Inflammatory Cytokines

**Figure 2:**
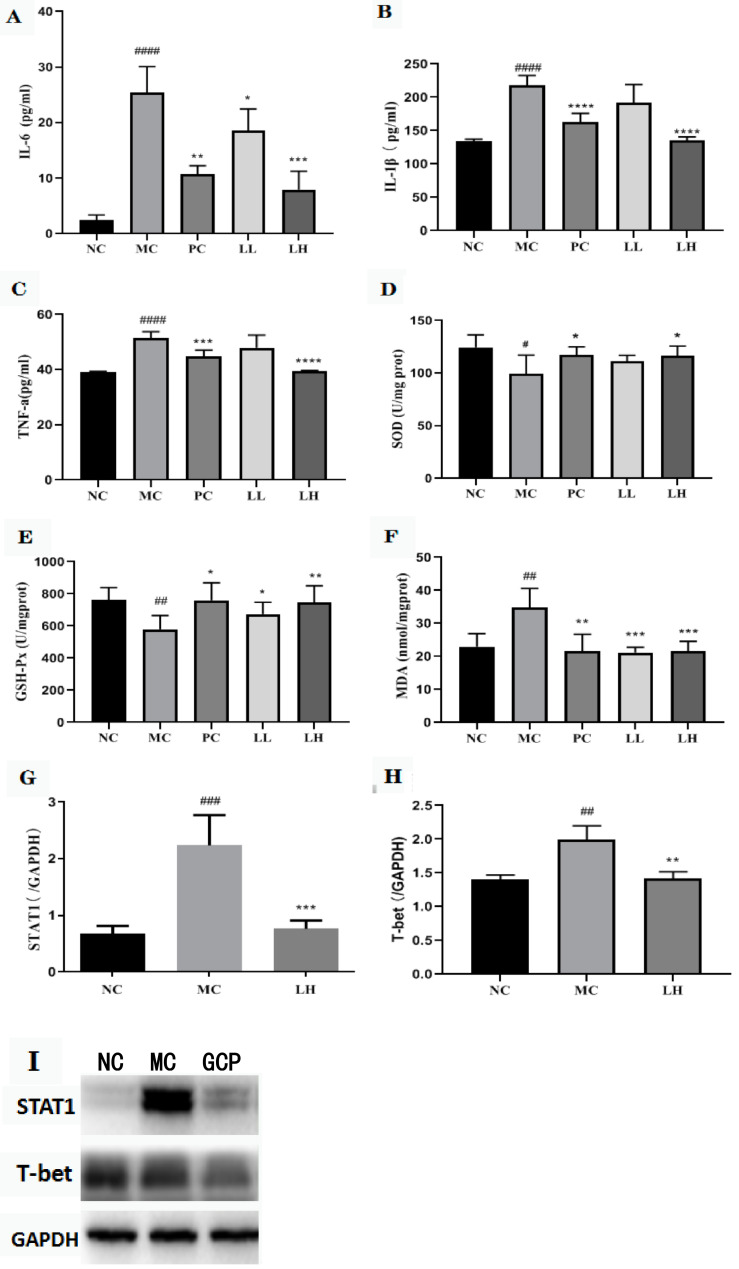
Effects of GCP on serum IL-6, IL-1*β*, and TNF-*α* levels. (**A**) Serum IL-6 levels in different groups. (**B**) Serum IL-1*β* levels in different groups. (**C**) Serum TNF-*α* levels in different groups. Effects of GCP on SOD, GSH-P_X_, and MDA levels. (**D**) The effect of GCP on serum SOD activities. (**E**) The effect of GCP on serum GSH-P_X_ activities. (**F**) The effect of GCP on serum MDA levels. Data are represented as mean ± SD, with n = 8 mice for each group. (**G**–**I**) Effects of GCP on the expression of STAT1 and T-bet. The presented values are the mean ± SD (n = 6 in each group) of three independent experiments. The density values of the Western blot were normalized for GAPDH. #### *p* < 0.0001, ### *p* < 0.001, ## *p* < 0.01, # *p* < 0.05, vs. control group, **** *p* < 0.0001, *** *p* < 0.001, ** *p* < 0.01, * *p* < 0.05, vs. CCl_4_ group.

At 16 h after CCl_4_-induced acute liver injury, a significant increase in the serum values of TNF-*α* and IL-6, IL-1*β* compared with the normal control (51.4 ± 3.1 and 25.36 ± 2.2 pg/mL, 217.72 ± 25 pg/mL, respectively) was observed. GCP group significantly reduced the release of TNF-*α*, IL-6, and IL-1*β* in the serum (47.95 ± 8.5, 18.53 ± 4.4, and 191.49 ± 28 pg/mL for 300 mg/kg; 39.36 ± 0.6, 7.95 ± 1.4, and 135.29 ± 7 pg/mL for 600 mg/kg). The biphenyl diester group significantly reduced the release of TNF-*α* and IL-6, IL-1*β* in the serum (44.9 ± 3.3, 10.69 ± 1.4, and 162.74 ± 25 pg/mL) (Figure 2A–C).

### 2.5. GCP Inhibited the Oxidative Stress Level

The CCl_4_-induced oxidative stress in mice liver was evaluated by assessing superoxide dismutase (SOD), glutathione peroxidase (GSH-Px), and malonic dialdehyde (MDA) levels. As shown in Figure 2D–F. CCl_4_ decreased the levels of SOD and GSH-Px in the livers by 1.25- and 1.32-fold, respectively, and increased the MDA level by 1.57-fold when compared to the normal control group, whereas mice in the GCP-pretreated group showed significant improvement towards the control values in a dose-dependent manner. GCP pretreatment at a high dose (600 mg/kg) increased the SOD and GSH-Px levels by 1.17- and 1.29-fold, respectively, and decreased the MDA level by 1.61-fold as compared with those values of the model control group.

### 2.6. Effect of GCP on STAT1 and T-Bet in CCl_4_-Induced Hepatitis

The total STAT1 protein and T-bet were analyzed to assess whether the activated STAT1/T-bet pathway could be abrogated by GCP prophylactic dosing. The model group significantly activated the expression of STAT1 protein after CCl4 stimulation, and then promoted the expression of T-bet protein (*p* < 0.01, *p* < 0.001). The expression of STAT1 and T-bet protein was significantly inhibited after GCP intervention (*p* < 0.01, *p* < 0.001) (Figure 2G–I).

### 2.7. Cell Viability of GNP and GAP

**Figure 3:**
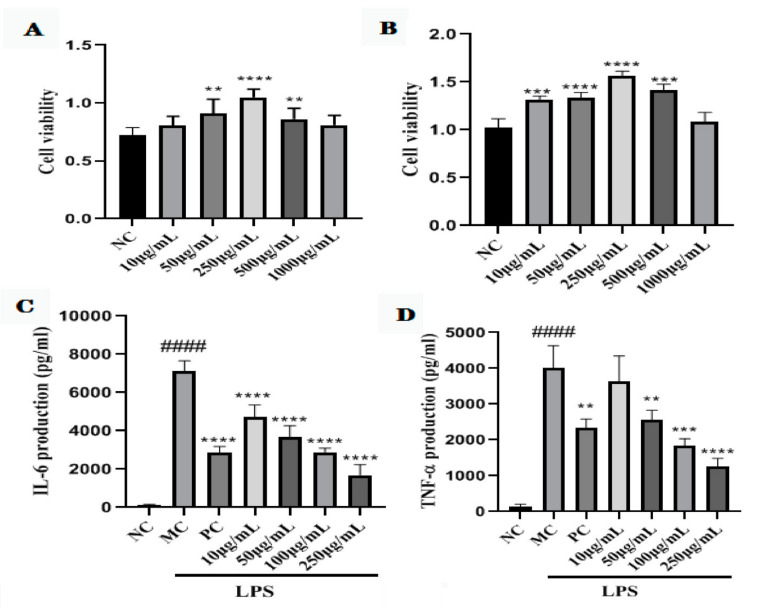

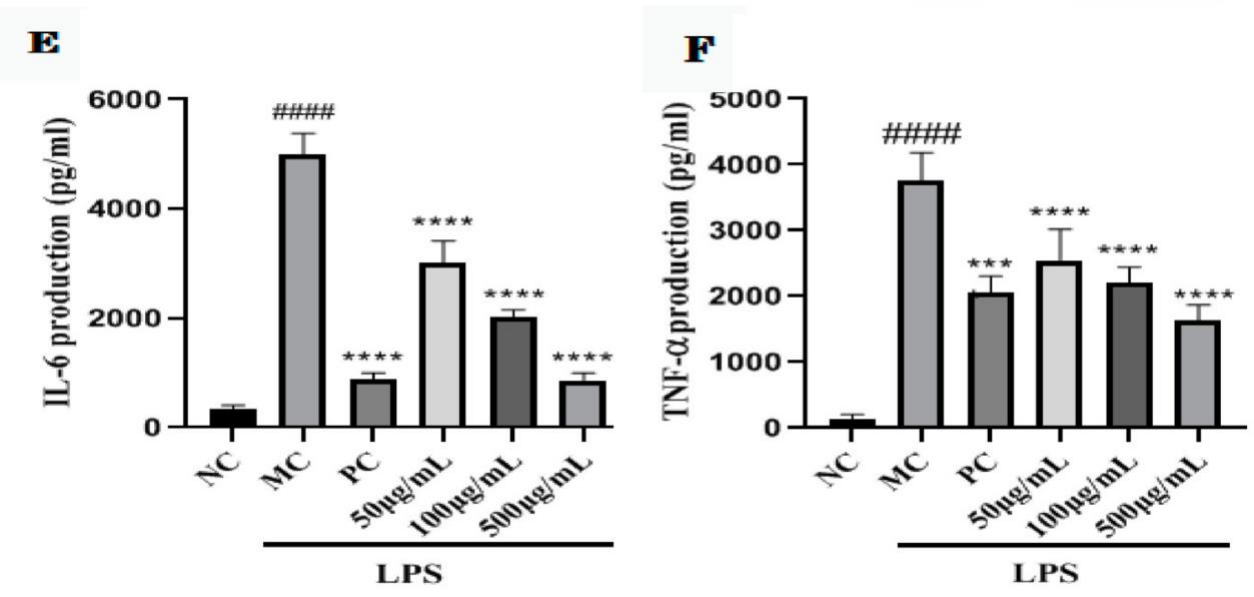
Cell viability map of GNP (**A**) and GAP (**B**); effects of GNP (**C**,**D**) and GAP (**E**,**F**) on serum IL-6 and TNF-*α* levels. Serum IL-6 levels in different groups (**C**,**E**). Serum TNF-*α* levels in different groups (**D**,**F**). #### *p* < 0.0001 vs. control group; ** *p* < 0.01, *** *p* < 0.001, **** *p* < 0.0001 vs. LSP group.

As shown in Figure 3A,B, all the concentrations of GCP did not show toxicity to the cells. Therefore, GNP and GAP at 50–500 μg/mL were used in the subsequent experiments.

### 2.8. GNP and GAP Inhibit the LPS-Activated Production of Pro-Inflammatory Cytokines in RAW 264.7 Macrophages

To further investigate the anti-inflammatory effect of GCP on the production of pro-inflammatory cytokines including TNF-*α* and IL-6, the RAW 264.7 macrophages were treated with LPS and GNP and GAP. Treatment with LPS alone notably induced TNF-*α* and IL-6 levels in RAW 264.7 macrophages (*p* < 0.0001). In contrast, GNP and GAP treatment suppressed the secretion of these LPS-stimulated pro-inflammatory cytokines in a concentration-dependent manner (*p* < 0.01, *p* < 0.001, *p* < 0.0001) (Figure 3C–F).

## 3. Materials and Methods

### 3.1. Materials and Reagents

The plant material of *G. wilfordii* was purchased from Kunming medicinal material market. The kits for the determination of ALT, AST, SOD, MDA, and GSH-Px were purchased from the Jiancheng Institute of Bioengineering. The ELISA kits for the determination of IL-1*β*, IL-6, and TNF-*α* were purchased from Link Bio. The STAT1 and T-bet rabbit polyclonal antibodies were purchased from American Proteintech. RAW264.7 macrophages were purchased from Beijing Beina Chuanglian Institute of Biotechnology. All other chemicals and reagents were of analytical grade.

### 3.2. Extraction and Purification of the Plant Polysaccharide

GCP was extracted from the dried aerial parts of *G. wilfordii* with hot water extraction and ethanol precipitation. The *G. wilfordii* crushed herbs were extracted 3 times with 10 vol of distilled water under reflux for 3 h (each time) at 80 °C. The combined aqueous extracts were filtered and concentrated under reduced pressure. Then added 80% EtOH solution to the extract, and the precipitated polysaccharides were collected by centrifugation (2000× *g*, 15 min, 20 °C). The precipitate was dissolved in distilled water and deproteinated by the method of Sevag [37]. Dissolved the precipitate in distilled water. Then, the concentrated solution was dialyzed (molecular weight cut-off at 3500 Da) in tap water for 48 h and then dialyzed in distilled water for 24 h and lyophilized to obtain crude polysaccharides (GCP). GCP was purified on DEAE Sephrose Fast Flow column (1.5 × 10 cm, i.d.), eluted the column with distilled water (1 mL/min), and 0.2 mol/L NaCl solution (1 mL/min), and monitored using the phenol-sulfuric acid method [38]. Finally, the purified polysaccharides (GNP and GAP) were collected, concentrated, and lyophilized for further study.

### 3.3. Structural Characterization of GCP, GNP, and GAP

#### 3.3.1. Determination of Polysaccharide and Protein Content

The content of GCP was determined by the phenol-concentrated sulfuric acid method [39], while the protein content was determined by the Coomassie brilliant blue method [40].

#### 3.3.2. Molecular Weight Determination

A high-performance gel-permeation chromatography (HPGPC) system (Agilent 1100, Santa Clara, CA, USA) with OHpak SB-803 HQ, Ohpak SB-804 HQ, Ohpak SB-805 HQ (8 × 300 mm) chromatographic column series, was used to measure GCP, GNP, and GAP molecular weights (MW). The column was eluted with 0.05 M NaCl solution at a flow rate of 0.6 mL/min. The temperature of the column and detector was 40 °C. The MW of GCP, GNP, and GAP was speculated based on the standard curve of the dextran series [41].

#### 3.3.3. Fourier Transform Infrared Spectroscopy (FT-IR)

The sample was ground with dried KBr and pressed into pellets for FT-IR measurement, repeated three times, and the detection range was 400–4000 cm^−1^ [30].

#### 3.3.4. Monosaccharide Composition

The monosaccharide composition of GNP and GAP were determined by ion chromatography. The polysaccharide was weighed and added to the TFA acid solution for continuous heating and hydrolysis for 2 h. After drying, it was put into a chromatographic bottle and eluted with a gradient of NaOH and NaOAc at a flow rate of 0.5 mL/min. The standard substance was determined by the same method [42,43].

#### 3.3.5. Methylation Analysis

The methylation method was performed to analyze the types of glycosidic bonds. After the polysaccharide was fully methylated, a full scan analysis and detection of the samples by GC-MS system were carried out [44].

#### 3.3.6. NMR Analysis

Next, 30 mg of GNP and GAP were dissolved in DMSO and D_2_O (0.5 mL, 99.9%) at 25 °C, to perform One-dimensional NMR (including ^1^H-NMR and ^13^C-NMR) and two-dimensional NMR spectra [36].

### 3.4. Animal Experiments

#### 3.4.1. Animal

The male Kunming mice (8-weeks-old) with a body weight (BW) of about 20 g were used in the present study. These were maintained in a room with a controlled temperature of 22 ± 0.5 °C, a normal day/night cycle, and allowed free access to a basal pellet diet and tap water. All procedures involving animals were conducted in accordance with the guidelines of China for the care and use of laboratory animals.

#### 3.4.2. Animal Grouping and Experimental Design

After a 7-day acclimatizing period, the mice were randomly distributed in five groups (eight mice each) including normal control, CCl_4_-induced acute liver injury (model group), bifendate (as positive control), and GCP groups. Mice in GCP groups were fed with GCP in two different doses (300 and 600 mg/kg BW per day for the low and high dose, respectively) by gastric gavage. In the same way, mice in the positive control group were fed with bifendate (150 mg/kg bw), while mice in normal and model control groups were given equal volume vehicles. All groups were fed once daily for 10 consecutive days. Except for the NC group, each group of mice was injected intraperitoneally (i.p.) with 1% CCl_4_ olive oil solution, 0.5 h after the final daily treatments. The mice in the NC group received an equivalent volume of olive oil (i.p.). All mice were sacrificed under anesthesia 16 h later.

### 3.5. Determination of Biochemical Indicators

The blood samples were collected immediately after the mice were sacrificed and centrifuged at 3000 rpm for 10 min at 4 °C to afford the serums. The serums were stored at 80 °C in the freezer. According to the procedure of kit instructions, the serum ALT and AST activities were measured. The serum TNF-*α*, IL-6, and IL-1*β* levels were determined using commercially available enzyme-linked immunosorbent assay (ELISA) kits [45].

The liver was excised, the liver samples were washed with cold saline solution to remove the blood, and dried with filter paper. Then, the liver was weighed, and the liver index was calculated according to the following formula.

$$\mathrm{liver}\;\mathrm{index}=\frac{\mathrm{liver}\;\mathrm{weight}\left(g\right)}{\mathrm{body}\;\mathrm{weight}\left(g\right)}\times 100\%$$

The liver tissue in an appropriate amount of ice saline was prepared into a homogenate containing 10% liver tissue. The homogenate was centrifuged at 3500 rpm and 4 °C for 15 min to obtain the supernatant. According to the kit instructions, NOS, SOD, and GSH-Px activities, and MDA levels in the liver homogenate were measured [46].

### 3.6. Histopathological Studies of Liver Tissues

The liver tissue at the same position of the right lobe of mice was taken and saline was used at 4 °C to wash away the residual blood on it and then dried over a filter paper. It was fixed with 10% neutral formalin, embedded with paraffin, sectioned, and stained with hematoxylin-eosin staining [47]. Histopathological changes in the liver tissue sections were observed under a light microscope.

### 3.7. Western Blotting

Collected liver and extracted its protein with RIPA reagent, then centrifuged at 3000× *g* for 10 min at 4 °C to get the supernatants. The protein was determined with a BCA kit in the supernatant. According to the molecular weight of the target protein, 10% and 12% of separation gel and 4% stacking gel were prepared. Electrophoretic parameters: stacking gel constant pressure 60 V, separation gel constant pressure 90 V. Transfer film parameters: constant electricity 200 mA, transfer time 1 h. The film was completely immersed in 5% BSA-TBST for 30 min on a shaker at room temperature, then incubated with primary antibody overnight at 4 °C. Washed 3 times with TBST, then the film was incubated with secondary antibody for 2 h on a shaker at room temperature. Washed 3 times with TBST, then the film was determined.

### 3.8. Cell Experiments In Vitro

#### 3.8.1. Cell Culture

The cells were incubated in DMEM supplemented with 10% FBS, 100 U/mL penicillin and 100 μg/mL streptomycin in a humidified atmosphere of 5% carbon dioxide (CO_2_) at 37 °C.

#### 3.8.2. Cell Viability Assay

The cell viability of GNP and GAP were measured by CCK-8 assay as described in previously study [48]. Briefly, the cells (1 × 10^5^ cells/well) were inoculated in 96-well plates for 24 h before being exposed to GNP (10–1000 μg/mL) and GAP (10–1000 μg/mL) in humidified 5% CO_2_ at 37 °C for 24 h, followed by the addition of reagent containing CCK-8 reagent (10 μL) in each well. After 1.5 h, the absorbance of each well was measured at 450 nm using a microplate reader.

#### 3.8.3. Determination of Pro-Inflammatory Cytokines Production

The RAW 264.7 macrophages were seeded into 96-well plates, pretreated with GNP (50–500 μg/mL), GAP (50–500 μg/mL), and 1 μM/L dexamethasone for 24 h, followed by the addition of LPS (0.1 μg/mL) for 24 h. The release of TNF-*α* and IL-6 in the culture supernatants was evaluated using ELISA kits.

### 3.9. Statistical Analysis

Data were expressed as mean ± standard deviation (S.D.). Student’s *t*-test and analysis of variance (ANOVA) were used to determine the statistical significance (Package for Social Sciences version 15.0, SPSS Inc., Chicago, IL, USA). A *p*-value less than 0.05 or 0.01 was considered significant.

## 4. Discussion

Polysaccharides are polymerized carbohydrate molecules, which have the advantages of high bioavailability, high solubility, and low toxicity. These are being used in the treatment of various diseases due to their wide range of biological activities, such as anti-inflammatory drugs. Their addition to the above biological activities, its remarkable hepatoprotective activity has attracted widespread attention. Different types of liver diseases such as non-alcoholic fatty liver disease and viral hepatitis have significant therapeutic or preventive effects [49].

According to literature reports, arabinogalactan, galactomannan, and pectin polysaccharides derived from higher plants have been shown to have significant antioxidant and anti-inflammatory activities [50,51]. The biological activities of most polysaccharides are closely related to their molecular weight, the way of glycosidic linkages and the composition of monosaccharides [52]. In this study, the molecular weight and infrared absorption of GCP were detected. In order to further clarify its structural characterization, the homogeneous polysaccharides GNP and GAP after separation and purification of GCP were hydrolyzed and derivatized to determine the composition of their monosaccharides and their connection method. The results showed that the molecular weight of GCP is approximately 1591 and 750.9 Da, GNP was mainly composed of Gal (32.3%), Glc (25.01%), and Ara (16.83%), while GAP was mainly composed of Ara (35.24%), Gal (26.33%), and Glc (23.48%). The main linking method of derivatives was →1)-Gal*p*-(4→. After clarifying its material basis, the mechanism of GCP’s hepatoprotective activity was explored in vitro and in vivo.

CCl_4_ is known to be a chemical with severe toxic effects on hepatocytes. The liver and kidney damage caused by carbon tetrachloride is mainly manifested in the free radicals generated in its metabolism. The mechanism is that CCl_4_ is activated by the liver microsome CytP450 to generate trichloromethyl free radicals (CCl_3_), which cause the peroxidation of unsaturated fatty acids in cell membranes and organelle membranes, thereby changing the fluidity and permeability of the membrane, inactivating the Ca^2+^-ATPase of the membrane, increasing the concentration of Ca^2+^ in the cytoplasm, destroying the cytoskeleton, activating phospholipase, and damaging amino acid functional groups, nucleic acid conversion, and mutation, and causing cell death. At the same time, the liver is induced to release the proinflammatory cytokines TNF-*α*, IL-6, and IL-1*β* following membrane lipid peroxidation [12], which directly lead to acute liver injury. In the present study, we demonstrated for the first time that GCP treatment can significantly prevent CCl_4_-induced acute hepatotoxicity in mice. The serum AST and ALT levels have been used as biochemical markers of acute liver injury [53]. Mice treated with CCl_4_ alone exhibited acute liver injury with significantly elevated serum AST and ALT levels compared with normal controls [54]. The serum AST and ALT significantly decreased after GCP treatment. In addition, the secretion levels of pro-inflammatory factors IL-6, IL-1*β*, TNF-*α,* and the content of MDA in CCl_4_ poisoned mice increased, and the content of SOD and GSH-Px decreased, resulting in liver damage. Continuous administration of GCP for 10 days significantly slowed down the inflammatory response and oxidative stress response in liver-damaged mice, which indicated a significant liver protective effect. At the same time, it was found by WB detection that the contents of STAT1 and T-bet protein in the liver of mice with liver injury decreased significantly after GCP intervention. According to literature reports, STAT1 can activate the transcription factor T-bet signaling pathway and plays an important role in the inflammatory response [55,56], so we determined that GCP also plays a key role in the STAT1/T-bet signaling pathway in hepatoprotective effect.

Macrophages are the most important inflammatory cells and play a key role in the inflammatory process by secreting a large number of pro-inflammatory mediators such as IL-6 and TNF-*α* [57]. The occurrence of inflammation is accompanied by the production of inflammatory mediators such as prostaglandin E2 (PGE2), IL-6, TNF-α, and nitric oxide (NO). The overexpression of these mediators may cause serious damage to cells and tissues, even resulting in organ failure [58,59]. CCl_4_-induced acute liver injury in mice has a sharp increase in the secretion of pro-inflammatory factors, and the excessive secretion of pro-inflammatory factors leads to further development of liver injury. In this study, lipopolysaccharide-activated macrophages RAW 264.7 were used to screen whether GNP and GAP have anti-inflammatory activity. Through experiments it was found that GNP and GAP can effectively reduce the secretion of pro-inflammatory factors, thereby slowing down the occurrence of inflammatory response and alleviating liver damage in mice.

## 5. Conclusions

The results showed that GCP has a significant protective effect on CCl_4_-induced acute liver injury in mice in vivo, and the protective effect of GCP on liver injury in mice was due to its inhibition of oxidative stress, inflammatory mediators, and STAT1/T-bet signaling pathway. In vitro, the homogeneous components of GNP and GAP also exhibited significant anti-inflammatory effects, and these pharmacological activities were closely related to their hepatoprotective effects.

## Figures and Tables

**Table 1:** Effects of GCP on liver and spleen index of CCl_4_ induced oxidative damage mice(n = 10).

Group	Mouse Liver Index**%** ± SD	Mouse Spleen Index**%** ± SD
Normal group (NC)	4.33 ± 0.27	0.43 ± 0.08
Model group (MC)	5.06 ± 0.14 ####	0.68 ± 0.9 ###
Positive control group (PC)	4.57 ± 0.34	0.51 ± 0.08 **
Low-dose group LL	4.37 ± 0.39 ***	0.63 ± 0.07 *
High-dose group LH	3.34 ± 0.27 ****	0.49 ± 0.04 **

Values are expressed as means ± SD of 10 mice in each group. #### *p* < 0.0001, ### *p* < 0.001, compared to the normal group. * *p* < 0.05, ** *p* < 0.01, *** *p* < 0.001, **** *p* < 0.0001, compared with CCl_4_-intoxicated group. Experimenters worked out the final results of hepatosomatic index (HI) in accordance with the subjacent expression: HI = liver weight/body weight ×100%.

## Supplementary Materials

The following supporting information can be downloaded at: https://www.mdpi.com/article/10.3390/molecules27113602/s1, Figure S1: HPGPC chromatogram of GCP (**A**), GNP (**B**), and GAP (**C**), 54 min is the solvent peak; the data of FT-IR on GCP (**D**), GNP (**E**) and GAP (**F**); total ion chromatograms of GNP (**G**) and GAP (**H**).; Figure S2: (**A**) ^1^H-NMR spectrum (600 MHz, DMSO, 30 °C); (**A**′) ^1^H-NMR spectrum (600 MHz, D_2_O, 30 °C); (**B**) ^13^C-NMR spectrum (150 MHz, DMSO, 30 °C); (**B**′) ^13^C-NMR spectrum (150 MHz, D_2_O, 30 °C); (**C**,**C**′) ^1^H/^13^C HSQC correlation spectrum; (**D**,**D**′) ^1^H-^1^H COSY correlation spectrum. Table S1: Methylation analysis data of GNP; Table S2: Methylation analysis data of GAP; Table S3: The ^1^H-NMR (600 MHz, DMSO) and ^13^C-NMR (150 MHz, DMSO) chemical shifts of GNP; Table S4: The ^1^H-NMR (600 MHz, DMSO) and ^13^C-NMR (150 MHz, DMSO) chemical shifts of GAP.

## Sample Availability

Samples of the compounds are available from the authors.
