# Supplementary material for: Hepatoprotective Polysaccharides from Geranium wilfordii: Purification, Structural Characterization, and Their Mechanism
Source: Molecules. 2022 Jun 3;27(11):3602. doi: 10.3390/molecules27113602 (PMC9182495; doi:10.3390/molecules27113602)
Supplement: Supplementary file 1 [file molecules-27-03602-s001.zip › molecules-1742480-supplementary.pdf]

Supplementary Materials

Table S1. Methylation analysis data of GNP.

Main methylation sugar	Type of linkage	Molar ratio (%)
2,3,5-Me3-Ara*f*	→1)-Ara*f*	8.5
2,3,4,6-Me-Glc*p*	→1)-Glc*p*	13.1
2,3-Me2-Ara*f*	→1)-Ara*f*-(5→	14.1
3,4,6-Me3-Man*p*	→1)-Man*p*-(2→	9.6
3,4,6-Me3-Glc*p*	→1)-Glc*p*-(2→	13.8
2,3,6-Me3-Gal*p*	→1)-Gal*p*-(4→	16.7
2,3,6-Me3-Glc*p*	→1)-Glc*p*-(4→	6.2

Table S2. Methylation analysis data of GAP.

Main methylation sugar	Type of linkage	Molar ratio (%)
2,3,5-Me3-Ara*f*	→1)-Ara*f*	8.78
3,4-Me2-Rha*p*	→1)-Xyl*p*-(2→	4.93
2,3,4,6-Me4-Glc*p*	→1)-Glc*p*	9.9
2,3,4,6-Me4-Gal*p*	→1)-Gal*p*	9.41
2,3-Me2-Ara*f*	→1)-Ara*f*-(5→	6.76
2,3,6-Me3-Gal*p*	→1)-Gal*p*-(4→	18.94
2,3,6-Me3-Gal*p*	→1)-Gal*p*-(4→	4.74
2,4-Me2-Gal*p*	→1)-Gal*p*-(3,6→	5.16

Table S3. The ^{1}H-NMR (600 MHz, DMSO) and ^{13}C-NMR (150 MHz, DMSO) chemical shifts of GNP

**Residue of GNP**	**Chemical shifts (ppm)**					
	**H1/C1**	**H2/C2**	**H3/C3**	**H4/C4**	**H5/C5**	**H6/C6**
A: →1)-*α*-D-Gal*p*-(4→	4.44/103.0	3.78/69.4	3.50/77.2	4.10/76.5	3.61/73.0	3.32/61.0
B: *α*-D-Glc*p*	5.19/92.1	3.80/74.2	3.51/74.3	3.24/71.1	3.66/73.0	3.35/61.1
C: →1)-*α*-D-Ara*f*	4.76/-	4.03/81.3	3.81/77.3	3.52/82.0	3.26/62.7	
D: →1)-*β*-D-Glc*p*	4.68/98.9	4.04/72.4	4.30/-	3.79/69.9	3.52/74.0	3.43/61.5
E: →1)-*β*-D-Glc*p*-(4→	4.48/-	3.16/73.5	3.62/72.9	3.45/81.7	-/74.3	3.77/61.4
F: →1)-*α*-D-Ara*f*-(5→	5.10/-	4.59/-	3.98/76.4	4.28/79.1	3.77/69.2	

-, not assigned

Table S4. The ^{1}H-NMR (600 MHz, DMSO) and ^{13}C-NMR (150 MHz, DMSO) chemical shifts of GAP

**Residue of GAP**	**Chemical shifts (ppm)**					
	**H1/C1**	**H2/C2**	**H3/C3**	**H4/C4**	**H5/C5**	**H6/C6**
A′: →1)-*β*-D-Glc*p*	4.33/103.6	3.32/74.3	3.53/76.7	3.72/72.5	3.57/74.3	3.70/61.0
B′: →1)-*α*-D-Ara*f*	5.00/107.0	3.94/81.5	3.80/76.0	3.58/80.7	3.44/61.6	
C′: →1)-*β*-D-Gal*p*-(4→	4.94/107.4	3.93/73.5	3.79/76.4	3.98/80.6	3.74/76.5	-/-

-, not assigned

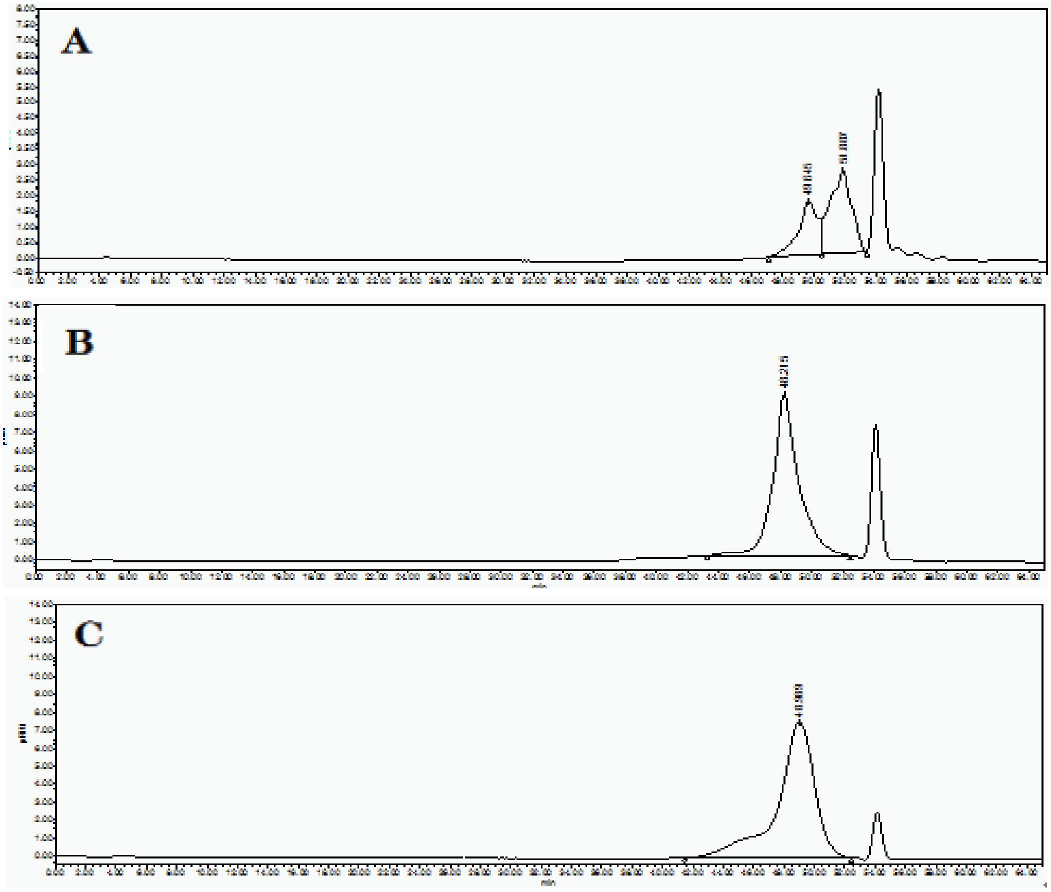
A
B
C

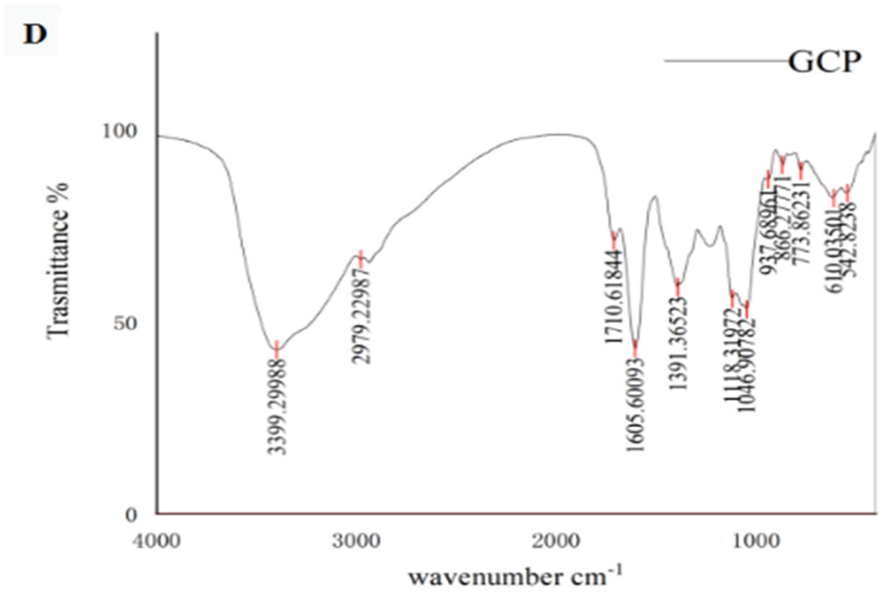
D
GCP
Trasmittance %
wavenumber cm-1
3399.29988
2979.22987
1710.61844
1605.60093
1391.36523
1118.31972
1046.90782
937.68961
866.7771
773.86231
610.03501
542.8238

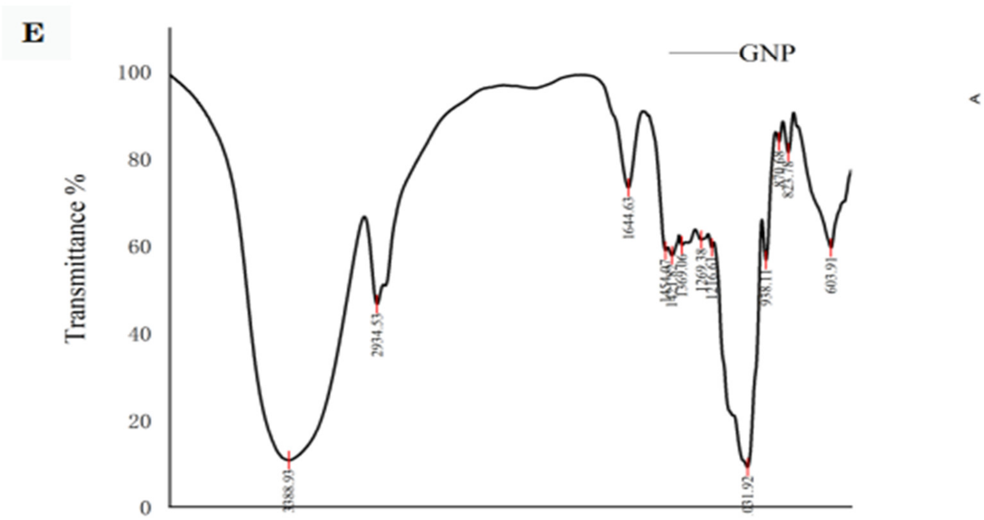
E
GNP
Transmittance %
3388.93
2934.53
1644.63
938.11
603.91

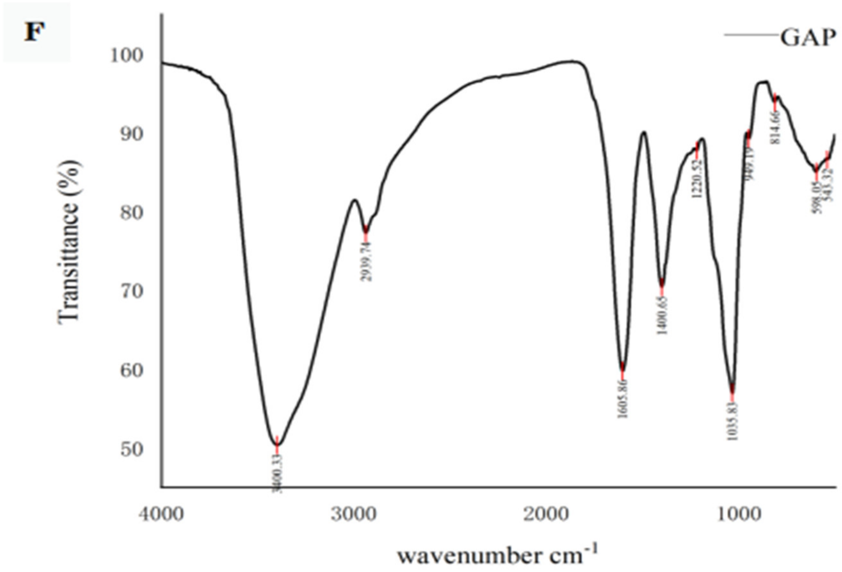
F
GAP
Transittance (%)
wavenumber cm-1
3400.33
2939.74
1605.86
1400.65
1220.52
1035.83
814.66

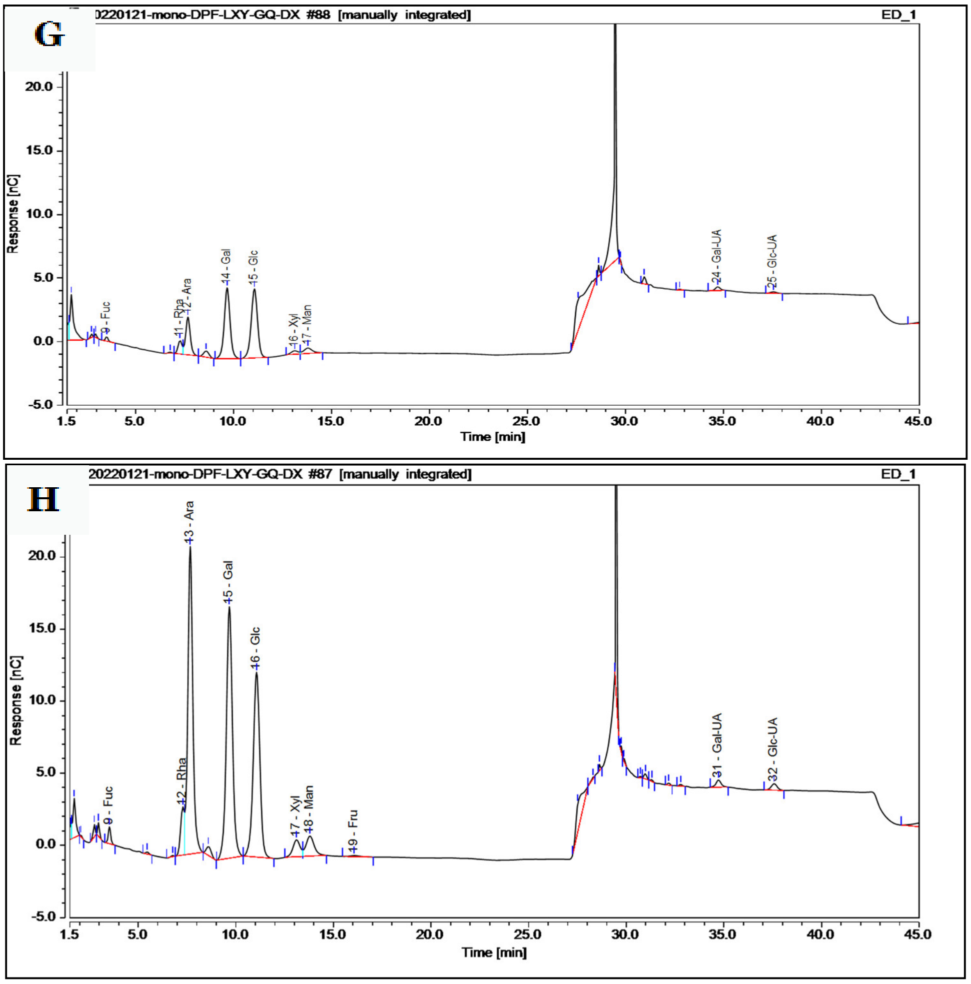

Figure S1. HPGPC chromatogram of GCP(A), GNP(B) and GAP(C), 54min is the solvent peak; The data of FT-IR on GCP(D), GNP(E) and GAP(F); Total ion chromatograms of GNP(G) and GAP(H).

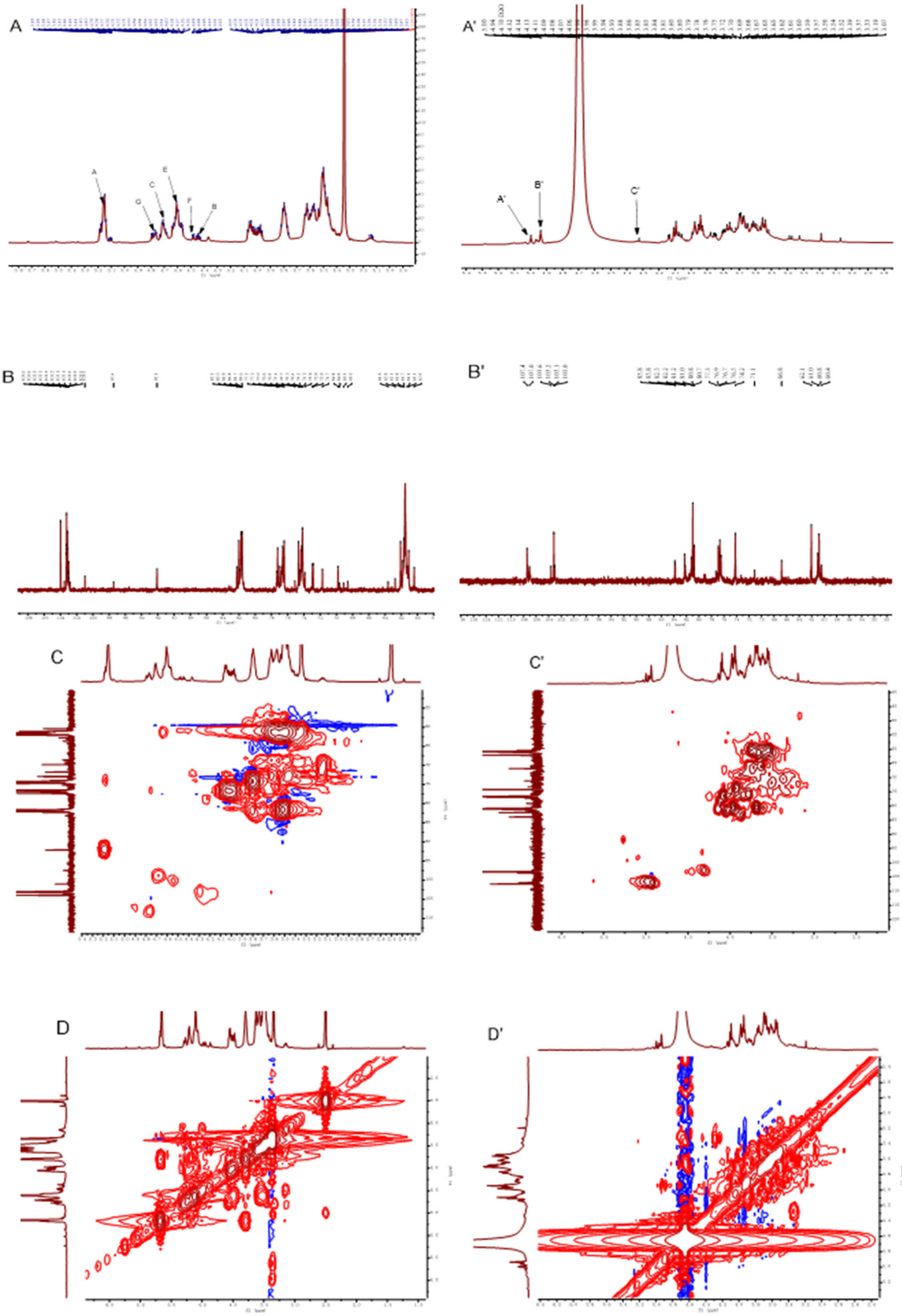

Figure S2. (A) ^{1}H-NMR spectrum (600 MHz, DMSO, 30°C); (A′) ^{1}H-NMR spectrum (600 MHz, D_2O, 30°C); (B) ^{13}C-NMR spectrum (150 MHz, DMSO, 30°C); (B′) ^{13}C-NMR spectrum (150 MHz, D_2O, 30°C); (C,C′) ^{1}H/^{13}C HSQC correlation spectrum; (D,D′) ^{1}H-^{1}H COSY correlation spectrum.
